# Randomised trials during a public health crisis, such as pandemic flu: a case study for a model of ‘off the shelf' ready-to-go trials

**DOI:** 10.1186/1745-6215-14-S1-P42

**Published:** 2013-11-29

**Authors:** Diane Whitham, Clare Brittain, Lelia Duley, Wei-Shen Lim

**Affiliations:** 1 University of Nottingham, Nottingham, UK; 2 Nottingham University Hospitals NHS Trust, Nottingham, UK

## Background

During the 2009 influenza pandemic NIHR funded a successful portfolio of studies. Nevertheless, the challenges of planning and conducting research during a pandemic were considerable. Clearly, advance planning would potentially have considerable advantages in terms of speed, efficiency and relevance of the research.

In 2011 the NIHR issued a themed call for pandemic flu research. Of 8 studies funded, 1 is a randomised trial comparing a short course of low dose steroids with placebo for adults admitted to hospital with influenza-like illness during pandemic flu. We present this as a case study for an ‘off the shelf' trial ready to be activated during a public health crisis. Study design and challenges will be presented and discussed.

## Methods

The trial aims to complete set-up, with approvals in place and IMP manufacture planned, by the end of 2013. It will then be hibernated, with annual review. Activation is when a pandemic is declared. Recruitment will be completed over six weeks, within the first wave of the pandemic, so results are available to inform practice during the second wave. Primary outcome is death or ICU admission, sample size is 2200. Follow up will be to 30 days.

## Results

A key issue is that the health service will be in a state of emergency. Challenges include drug and placebo readiness, R&D approvals, maintaining awareness.

## Discussion

This model has potential for wide application to public health crises. Successful conduct of trials in these situations would improve preparedness and response in public health emergencies.

